# Hepatitis B Virus-Associated Hepatocellular Carcinoma and Hepatic Cancer Stem Cells

**DOI:** 10.3390/genes9030137

**Published:** 2018-03-02

**Authors:** Saravana Kumar Kailasam Mani, Ourania Andrisani

**Affiliations:** Department of Basic Medical Sciences and Purdue Center for Cancer Research, Purdue University, West Lafayette, IN 47907, USA; skailasa@purdue.edu

**Keywords:** Hepatitis B Virus (HBV), Hepatocellular Carcinoma (HCC), hepatic Cancer Stem Cells (hCSCs), Epigenetics, Polycomb Repressive Complex 2 (PRC2), Epithelial Cell Adhesion Molecule (EpCAM), pluripotency genes, p68/DDX5 RNA helicase, Hox transcript antisense RNA (*HOTAIR*)

## Abstract

Chronic Hepatitis B Virus (HBV) infection is linked to hepatocellular carcinoma (HCC) pathogenesis. Despite the availability of a HBV vaccine, current treatments for HCC are inadequate. Globally, 257 million people are chronic HBV carriers, and children born from HBV-infected mothers become chronic carriers, destined to develop liver cancer. Thus, new therapeutic approaches are needed to target essential pathways involved in HCC pathogenesis. Accumulating evidence supports existence of hepatic cancer stem cells (hCSCs), which contribute to chemotherapy resistance and cancer recurrence after treatment or surgery. Understanding how hCSCs form will enable development of therapeutic strategies to prevent their formation. Recent studies have identified an epigenetic mechanism involving the downregulation of the chromatin modifying Polycomb Repressive Complex 2 (PRC2) during HBV infection, which results in re-expression of hCSC marker genes in infected hepatocytes and HBV-associated liver tumors. However, the genesis of hCSCs requires, in addition to the expression of hCSC markers cellular changes, rewiring of metabolism, cell survival, escape from programmed cell death, and immune evasion. How these changes occur in chronically HBV-infected hepatocytes is not yet understood. In this review, we will present the basics about HBV infection and hepatocarcinogenesis. Next, we will discuss studies describing the mutational landscape of liver cancers and how epigenetic mechanisms likely orchestrate cellular reprograming of hepatocytes to enable formation of hCSCs.

## 1. Hepatitis

Hepatitis is inflammation of the liver due to metabolic (toxins, drug, alcohol), genetic, autoimmune, ischemic, infection (virus, parasite, bacteria), and other reasons [1,2,3,4,5,6,7,8]. Among them, viral hepatitis is the most common type of hepatitis worldwide [6]. All five hepatitis viruses (A, B, C, D and E) cause inflammation of the liver. Hepatitis A and E viruses usually cause acute hepatitis while hepatitis B, C and D viruses could cause either acute or chronic hepatitis [9]. Particularly, hepatitis B virus (HBV) and hepatitis C virus (HCV) infection could cause chronic inflammation leading to liver cancer or hepatocellular carcinoma (HCC) [9].

## 2. The HBV Life Cycle

HBV is a non-cytopathic, partially double-stranded hepatotropic DNA virus, belonging to the hepadnaviridae family. The 3.2 kb HBV genome encodes four overlapping open reading frames [10,11,12] (Table 1). 

**Figure 1:**
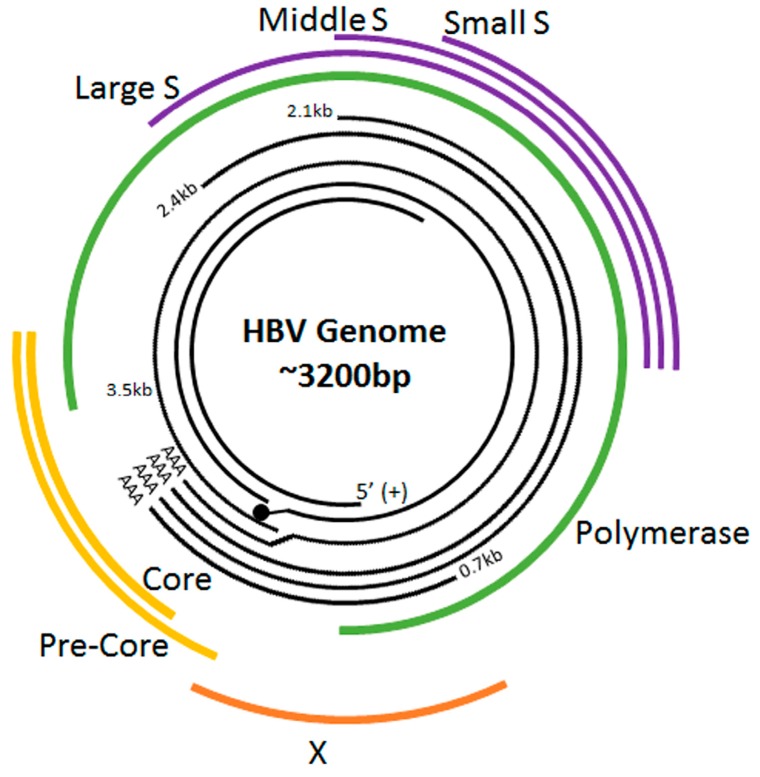
Hepatitis B Virus (HBV) Genome Organization. The innermost two black lines represent the full-length minus (–) strand (with the terminal protein attached to its 5′ end) and the incomplete plus (+) strand. The outer black lines represent the 3.5, 2.4, 2.1 and 0.7 kb mRNA transcripts. The outermost lines indicate the translated HBV proteins.

**Figure 2:**
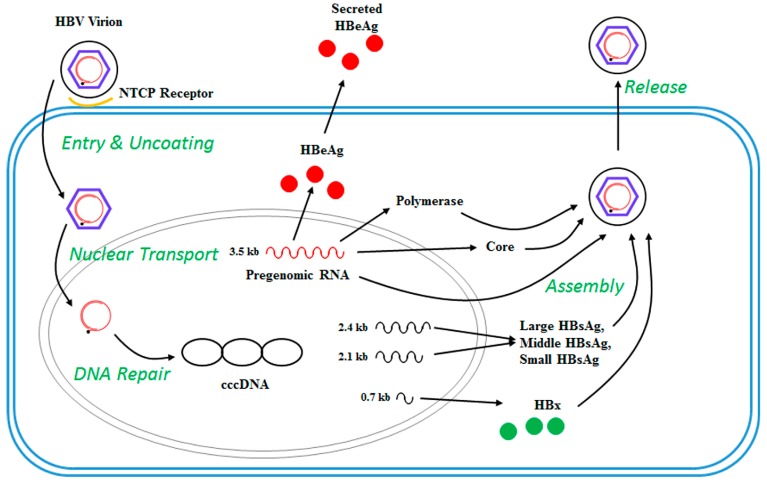
HBV Life Cycle. HBV uses the Sodium Taurocholate Co-transporting Polypeptide (NTCP) receptor to attach to hepatocytes. After entry, HBV nucleocapsids transport the HBV DNA to the nucleus, where the relaxed circular DNA is converted into covalently closed circular (ccc) DNA. The cccDNA assumes a minichromosome-like structure and acts as the template for transcription of four viral RNAs (0.7 kb, 2.1 kb, 2.4 kb and 3.5 kb). The mRNA transcripts are exported to the cytoplasm and used for translation of the HBV proteins. The longest (pre-genomic) RNA also functions as the template for replication, which occurs within nucleocapsids in the cytoplasm. Nucleocapsids are enveloped during their passage through the endoplasmic reticulum (ER) and/or Golgi complex and secreted from the cell. HBeAg: Hepatitis B e antigen; HBsAg: Hepatitis B surface antigen, HBx: Hepatitis B X protein.

Hepatitis B e antigen (HBeAg) is a circulating peptide derived by proteolytic processing of the pre-core protein encoded by gene C that is then modified and secreted from liver cells. It usually serves as a marker of active viral replication. The HBsAg gene is one long open reading frame that contains three in frame “start” (ATG) codons which divide the gene into three sections, pre-S1, pre-S2, and S. Because of the multiple start codons, three polypeptides are produced [13] referred to as large hepatitis B surface antigen (LHBsAg) containing pre-S1, pre-S2, and S; middle hepatitis B surface antigen (MHBsAg) containing pre-S2, S and small hepatitis B surface antigen (SHBsAg). The LHBsAg, MHBsAg and SHBsAg envelope proteins associate with the endoplasmic reticulum (ER) membrane as part of their replication process [14]. A schematic of the HBV genomic structure [15] and life cycle [15] is shown in Figure 1 and Figure 2, respectively.

## 3. Chronic Viral Hepatitis B and Hepatocellular Carcinoma (HCC)

An estimated 257 million people are currently chronically infected with HBV [6]. About 887,000 die every year due to HBV complications [16]. Several mechanisms by which chronic HBV infection leads to cancer have been identified. Following HBV infection, a robust T-cell immune response is elicited to combat the infection. This results in hepatocyte necrosis, inflammation and consequently regeneration, to compensate for lost hepatocytes [15,17,18]. When the immune system fails to clear HBV, there are sustained cycles of necrosis-inflammation-regeneration [17]. Such continuous proliferation of hepatocytes likely enables propagation of epigenetic alterations, oncogenic mutations, and telomere shortening with consequent genomic instability. 

In addition to chronic inflammation, another mechanism through which HBV contributes to hepatocarcinogenesis is via viral protein-endoplasmic reticulum (ER) interactions [19]. Specifically, interaction of LHBsAg, MHBsAg and SHBsAg proteins with the host ER induces ER stress leading to induction of oxidative stress [20]. This stimulates growth and survival signaling pathways, causes mutations through the generation of free radicals, and activates stellate cells [17,21]. Furthermore, the HBV genome is found integrated in the host genome in nearly all HBV-associated liver tumors, likely contributing to hepatocyte transformation. Mechanisms by which the integration of HBV DNA could contribute to hepatocarcinogenesis include host DNA alterations at several cancer-relevant genes including cyclin A, telomerase reverse transcriptase (*TERT*), platelet-derived-growth-factor receptor-beta (*PDGFRB*), mitogen activated protein kinase 1 (*MAPK1*) and others [22,23]. 

Furthermore, the HBV encoded HBx protein, a 16.5 KDa protein which is essential for the viral life cycle, activates cellular mitogenic signaling cascades and their downstream transcription factors including NF-kB, AP-1, AP-2, c-EBP, ATF/CREB, thereby altering expression of growth-control genes [24,25,26,27,28]. Significantly, Terradillos et al. showed that although transgenic mice expressing HBx in the liver did not develop liver tumors, HBx/c-Myc bi-transgenic mice expressing c-Myc under the control of the Woodchuck Hepatitis Virus regulatory elements, exhibit accelerated formation of liver tumors by 2–3 months [29]. Whether HBx accelerates tumor formation by inhibiting DNA repair or promoting cell cycle progression was studied by Madden et al. [30]. Transgenic mice expressing HBx (ATX mice) and having an integrated lambda transgene (to measure mutation frequency) were treated with DEN (Di-Ethyl-Nitrosamine) to induce DNA damage. DEN-treated ATX mice developed 70% more expansible, basophilic, focal lesions than DEN-treated wild-type mice, without a significant increase in the accumulation of DNA mutations. These results demonstrated that HBx did not affect DNA repair following DEN-induced DNA damage. Importantly, the rates of hepatocyte proliferation, measured by immunohistochemical detection of proliferating cell nuclear antigen (PCNA) and by 5-bromo-2′-deoxyuridine (BrdU) incorporation, were significantly increased in livers of ATX mice. Accordingly, it was concluded that HBx contributes to development of DEN-induced liver cancer by promoting proliferation of “altered cells” rather than by inhibiting DNA repair [30]. 

In vitro studies have also provided mechanistic insight towards understanding HBV-mediated hepatocarcinogenesis [31]. Specifically, HBx expressing cells exhibited increased CDT1 (a DNA replication licensing factor, required for pre-replication complex assembly) as well as reduced geminin expression (a protein that inhibits DNA replication by preventing the incorporation of Mini Chromosome Maintenance complex into the pre-replication complex). This altered expression of CDT1 and geminin results in an increased CDT1 to geminin ratio, allowing DNA re-replication in the G2 phase of HBx expressing cells [31]. Moreover, HBx expressing cells in the G2 phase activate ATR signaling, indicative of replication stress, without activation of the DNA damage checkpoint kinase CHK1. In support of this observation, it was found that in HBx expressing cells, the expression of the mitotic kinase PLK1 which mediates recovery from G2/M checkpoint was also elevated [32]. Significantly, concurrent staining of phospho-Histone 3 and γH2AX in the G2/M phase of HBx-expressing cells indicated propagation of DNA damage, due to DNA re-replication, to daughter cells [31]. Continued propagation of DNA damage to daughter cells eventually results in oncogenic transformation which can be suppressed by PLK1 inhibition, as was shown in an in vitro cellular system model [33] and is also supported by in vivo animal studies [34]*.* Based on these results, it is now well-accepted that HBx acts as a co-factor in hepatocarcinogenesis. 

## 4. Mutational Landscape of HBV-Related HCC

Using whole genome and whole exome sequencing, researchers have analyzed the genetic landscape of HCCs [35,36,37,38,39]. Unlike most solid tumors, multiple mutations with significant incidence have been observed in HCC. This heterogeneity of HCC is unlike other cancers, e.g., pancreatic and lung cancers characterized by activating Ras^V12^ mutations. Accordingly, molecular classification and biomarker identification of HCC is challenging. The majority of the genomic studies performed to date have analyzed HCC samples of different etiologies, i.e., due to chronic infection by HBV and HCV, alcohol abuse, metabolic syndrome etc. As the number of tissue samples analyzed increase, specific mutational patterns emerge, linked to tumor etiology. 

Recent studies observed that single nucleotide polymorphisms (SNPs) of genes including *GSTM1* (Glutathione S-Transferase Mu1), *GSTT1* (Glutathione S-Transferase Theta1), *STAT4* (Signal Transducer and Activator of Transcription 4), *TPTE2* (Transmembrane Phosphoinositide 3-Phosphatase and Tensin Homolog 2), *DCL1* (CD302 Molecule), *KIF1B* (Kinesin Family Member 1B) and *PGD* (Phosphogluconate Dehydrogenase) are associated with increased risk of HBV-mediated HCC [40]. Totoki et al. analyzed 503 liver cancer genomes (117 HBV positive cases) from different countries to identify candidate driver genes [35]. It was observed that HBV-infected patients displayed *TERT* promoter mutations, *TERT* gene amplification, which rarely co-occurred with HBV integration in the *TERT* locus. Also, *AXIN1* was found to be more frequently mutated in HBV positive than in HCV positive or non-virus HCC cases. In another study, Guichard et al., performed copy number analysis in 125 HCC tumors and whole exome sequencing of 24 tumors [36]. They found that highly rearranged copy number profiles were more frequent in HBV HCC tumors as compared to HCC tumors of other etiology. Also, IRF2 (Interferon Regulatory Factor 2) which interacts with MDM2 and plays key role in cell growth regulation and immune response was mutated in ≈17% (6 out of 35) of HBV-associated liver tumors.

Moreover, mutations in the tumor suppressor p53 (*TP53*), WNT pathway (*APC*, *AXIN1*, *CTNNB1*), telomere maintenance (TERT), and epigenetic enzymes (*ARID1A*, *ARID2*, *MLL4*) have also been reported in HBV-mediated HCC [40,41]. Some of these mutations occur due to HBV insertion, although it is not understood what causes mutations in other instances. Repeated cycles of cell proliferation and cell death during inflammation are likely responsible for the genesis of the above described mutations. Alternatively, HBV infection may alter the chromosomal architecture or genome topology of the infected hepatocyte, an area of study that remains to be explored and better understood. More importantly, how these mutations (excluding p53 and WNT signaling) contribute to the mechanism of HBV-related HCC pathogenesis remains to be understood.

Recently, epigenetic mechanisms initiated by HBx have been shown to be involved in HBV mediated HCC, and importantly, these studies also provided evidence that these epigenetic mechanisms have a role in virus biosynthesis [42]. The template of viral transcription in the infected hepatocyte nucleus is cccDNA, which associates with histones forming chromatin-like structure, the viral minichromosome [43]. Accordingly, the accessibility of the HBV cccDNA to regulatory transcription factors, based on the histone modifications associated with the HBV minichromosome, determines the rate of viral transcription. Indeed, acetylation of histones associated with the HBV cccDNA/minichromosome resulted in increased HBV replication in a HepG2 cell-based model [44], while methylation of HBV cccDNA reduced viral gene expression [45]. Accordingly, epigenetic mechanisms deregulated by HBV infection likely contribute to both the regulation of virus biosynthesis and HCC pathogenesis. However, more in depth mechanistic studies are needed to prove this connection.

## 5. Epigenetic Mechanisms in HBV-Related HCC

Epigenetic modifications are heritable changes in gene expression that do not result from changes in the genomic sequence. These may be due to: (i) DNA modifications (methylation of cytosine residues generating 5-methylcytosine, oxidation of 5-methylcytosine to 5-hydroxymethylcytosine); (ii) Histone modifications (methylation, acetylation, phosphorylation, and ubiquitination of N-terminal of histone tails); (iii) Nucleosome re-structuring by ATP-dependent chromatin remodeling complexes, and (iv) altered expression of long non-coding RNAs (lncRNA) and microRNAs (miRNAs). 

Environmental factors (life style, diet, viral infections) are proposed to be the main drivers of epigenetic changes. These epigenetic changes are required for normal development of an organism as well as cellular functions. However, abnormal epigenetic changes could also lead to disease pathogenesis. Epigenetics is involved in the initiation, progression and metastasis of liver cancer [46,47]. All four categories of epigenetic modifications have been identified in liver cancer (Table 2). 

Since epigenetic mechanisms are involved in developmental programming of normal stem cells to specific lineages during cellular differentiation [59,60], deregulation of epigenetic mechanisms may also cause loss of differentiation ability and acquisition of stem cell-like characteristics during cancer pathogenesis [61]. These stem-like cells are termed cancer stem cells (CSCs) because they exhibit characteristics including extensive self-renewal, expression of pluripotency genes, altered expression of genes involved in cellular metabolism, in cell cycle progression and survival mechanisms, and potent tumor initiating potential [61,62,63]. How all of these cellular changes are orchestrated during oncogenic transformation leading to formation of CSCs is not fully understood [64,65]. For example, c-Myc, which is frequently overexpressed in liver cancer [66], can regulate pluripotent stem cell fate through regulation of metabolic flux [67]. Signaling pathways including Wnt, Hedgehog, and Notch also modulate the stem cell state via induction of their downstream target genes through epigenetic regulation [68,69,70]. Hence, aberrant epigenetic modifications could deregulate a variety of cellular processes to promote cancer progression, but the detailed mechanisms remain to be deciphered.

Pluripotent cells have the ability to self-renew and differentiate into all cell types in the body. Similar to a normal stem cell, cancer stem cells have been discovered in several cancers [71,72,73,74] including liver cancer [75]. CSCs are considered pluripotent in their ability to self-renew and give rise to diverse tumor cells Thus, CSCs contribute to tumor heterogeneity and tumor relapse after chemotherapy; however, mechanisms resulting in the formation of CSCs are incompletely understood. Two main models have been proposed regarding the cellular origin of CSCs: (i) CSCs are formed from normal stem cells/or tissue progenitors due to mutations, epigenetic changes, and other mechanisms; (ii) CSCs are formed due to dedifferentiation/cellular reprogramming of differentiated somatic cells. Widschwendter et al. provided evidence supporting the former model [76]. Embryonic stem cells rely on Polycomb group proteins to reversibly repress genes required for differentiation. These Polycomb gene targets are up to 12-fold more likely to have cancer-specific promoter DNA hypermethylation than non-targets, supporting a stem cell origin of cancer [76]. Specifically, reversible gene repression, replaced by permanent silencing, e.g., DNA methylation, locks the cell into a malignant stem cell-like state. However, other researchers have provided evidence supporting the latter model. 

Regarding liver cancer and the cellular origin of hCSCs, in vivo studies employing mouse hepatic progenitor cells, lineage-committed hepatoblasts, and differentiated adult hepatocytes transduced with transgenes encoding H-RAS and SV40LT acquired markers of CSCs and self-renewal ability, and formed tumors in mice [77]. These studies clearly established two important points; first, every hepatic lineage cell is susceptible to oncogene-driven transformation, and second, every hepatic lineage cell could develop features characteristic of hCSCs, resulting in an aggressive phenotype [77]. Other studies also support the de-differentiation of adult hepatocytes, for example, via loss of p53 or overexpression of *YAP1*, into progenitor-like cells capable of malignant transformation [78,79]. Lineage tracing studies in mice showed that following liver injury, differentiated hepatocytes reprogram to a distinct cell population resembling hepatic progenitors [80,81]. Human hepatocytes also have the same capacity [80]. Since HBV biosynthesis requires the transcription factor HNF4α expressed in differentiated hepatocytes [82], we reason that chronic HBV infection must promote the reprogramming of differentiated hepatocytes to hepatic-like progenitors or hCSC during liver cancer pathogenesis, by a mechanism not fully understood. This idea is supported by recent studies demonstrating resistance of stem cells to viral infection [83], which would exclude HBV infection of hepatic progenitors. 

Thus, the question is how does chronic HBV infection mediate the cellular reprogramming of the differentiated hepatocyte to hCSCs? From the study of mouse embryonic fibroblasts, the epithelial cell adhesion molecule (EpCAM) and its associated protein Claudin-7 were shown to promote pluripotency reprogramming through upregulation of pluripotency transcription factors and repression of p53 and p21 expression [84]. Interestingly, *EPCAM* is repressed by the chromatin modifying PRC2 complex [85], suggesting that loss of PRC2 function has a role in this cellular reprogramming, as has recently been shown for therapy-resistant leukemias with PRC2 inactivation [86]. 

## 6. Polycomb Repressive Complex 2 (PRC2)

PRC2 consists of three core subunits, SUZ12, EZH2, and EED, mediating the trimethylation of lysine 27 on Histone 3 (H3K27me3), which represses gene expression [87]. It is involved in lineage selection during embryogenesis. EZH2 is required to generate mesodermal lineages [88], while *SUZ12* knockout (KO) ESCs fail to generate proper endodermal lineages [89] such as hepatocytes. Surprisingly, *EED* KO ESCs are able to differentiate into the three germ layers [90]. PRC2 is also essential for embryonic development. *Eed*^−/−^, *Ezh2*^−/−^, and *Suz12*^−/−^ mouse embryos display severe defects during gastrulation [89,91,92]. Chromatin modifiers, including polycomb repressor complexes, also target metabolic enzymes within their active gene sets [93,94]. Furthermore, EZH2, the methyltransferase component of PRC2, can function independently of the other PRC2 subunits, methylating non-histone proteins [95]. For instance, EZH2 activates NF-κB target genes in breast cancer [96], and in a subpopulation of glioblastoma stem cells, EZH2 methylates STAT3, leading to enhanced STAT3 activation [97]. EZH2 also exhibits PRC2-independent functions in prostate cancer, where it acts as co-activator for critical transcription factors including the androgen receptor [95]. 

EZH2 and its associated PRC2 complex are the most significantly deregulated epigenetic regulators in primary HCC samples [98]. Increased expression of EZH2 in HCC results in activation of Wnt signaling by silencing Wnt antagonists [99]. Wnt signaling is one of the key pathways that contribute to expression of pluripotency genes. Interestingly, although EZH2 is overexpressed in most subtypes of HCC, SUZ12, another core subunit of PRC2 is downregulated at the protein level in certain subtypes of HCC, including poor prognosis HBV-mediated HCCs [56,85]. The HBx protein encoded by HBV, essential for the viral life cycle, activates the cellular mitogenic PLK1 kinase [100,101]. In turn, activated PLK1 induces proteasomal degradation of SUZ12 by site-specific phosphorylation [55]. PLK1-dependent ubiquitination of SUZ12 is enhanced by overexpression of HOTAIR [56], which serves as an ubiquitination scaffold in association with RNA binding E3 ubiquitin ligases [102]. Since murine *Suz12*^−/−^ embryonic stem cells are characterized by global loss of H3K27me3 [103], downregulation of SUZ12 protein levels in HBV infected cells could be linked to formation of hCSCs. In HBx-expressing model cell lines, *SUZ12* knockdown results in re-expression of *EPCAM* and pluripotency genes [56], and a hCSC-like gene signature was found elevated in poor prognosis HBV-associated HCC [54]. Interestingly, these HBV-associated HCCs overexpressed EZH2 [54], suggesting that in addition to the downregulation of SUZ12, enhanced expression of EZH2, acting independently of PRC2, could also contribute to Wnt activation by silencing Wnt antagonists [99].

## 7. Epithelial Cell Adhesion Molecule (EpCAM) and Wnt Activation

EpCAM is a transmembrane glycoprotein involved in cell signaling, migration, proliferation, and differentiation [104,105,106,107]. *EPCAM* is highly expressed in carcinomas, tumor-initiating cells, tissue progenitor cells, embryonic, and adult stem cells, but at lower levels in normal epithelia [108,109,110]. *EPCAM* is expressed in hepatic progenitors [111] and hCSCs [112]. Human EpCAM, comprised of 314 amino acids (aa), consists of an N-terminal extracellular domain (EpEX) of 242 aa, a transmembrane domain of 23 aa, and a C-terminal cytoplasmic domain (EpICD) of 26 aa [113]. EpCAM undergoes regulated intramembrane proteolysis via the TACE enzyme, resulting in EpEX release in the extracellular microenvironment, and EpICD release in the cytoplasm [106]. EpICD forms a complex with FHL2, β-catenin, and transcription factor Lef1, promoting transcription of β-catenin-regulated genes [106,114], including *EPCAM*, *NANOG*, *OCT4*, *KLF4*, *SOX2*, and *MYC* [115] involved in cell cycle regulation and stemness.

Yamashita et al. isolated EpCAM positive cells from HCC cell lines and generated evidence that EpCAM positive HCCs are hCSCs [112]. Also, our work demonstrated that increased *EPCAM* expression, activation of Wnt signaling, and expression of pluripotency genes occur in a subpopulation of HBV replicating cells exhibiting significantly reduced SUZ12 protein levels and properties of hCSCs [54]. Importantly, increased *EPCAM* and pluripotency gene expression was quantified by RT-PCR in a cohort of HBV-related HCCs that exhibited poor prognosis after tumor resection [54]. This increased *EPCAM* and pluripotency gene expression positively correlated with reduced expression of another epigenetic regulator, the RNA helicase DDX5 [42].

## 8. P68 (DDX5)

DDX5 is a DEAD-box (Asp-Glu-Ala-Asp) family RNA helicase with RNA dependent ATPase activity [116,117]. It is important for pre-mRNA, rRNA and miRNA processing [118]. It can also act as a co-activator for transcription factors including estrogen receptor-alpha, androgen receptor, MyoD, RUNX2 and p53 [119,120,121,122,123]. Abnormal expression of DDX5 has been reported in colon, breast, prostate, leukemia, glioma and HCC [120,124,125,126,127,128]. Interestingly, although increased expression of DDX5 is observed in other cancers, it is downregulated in HBV-associated HCC [42,128]. DDX5 has putative seed sequences of microRNAs (miRNAs) belonging to miR-106b~25 and miR-17~92 clusters which are upregulated in HBV-HCC [52]. Thus, increased expression of these miRNAs is likely responsible for the observed downregulation of DDX5 in subtypes of HCC [129]. We have shown that DDX5 stabilizes SUZ12, a component of PRC2 and protects it from proteasomal degradation [42]. Hence, downregulation of DDX5 by miRNAs could render SUZ12 susceptible to degradation, thereby reducing PRC2 activity and allowing re-expression of key oncogenes.

## 9. Hox Transcript Antisense RNA (*HOTAIR*)

*HOTAIR* is a 2.2 kb lncRNA encoded by the HOXC locus of the HOX gene cluster [130]. *HOTAIR* recruits PRC2 to repress expression of genes of the HOXD locus [131]. In addition to epigenetic repression of genes via PRC2, *HOTAIR* also functions as competing endogenous RNAs (ceRNAs) to sponge miRNAs, thereby regulating derepression of miRNA targets [132]. In breast cancer, *HOTAIR* reprograms the PRC2 binding profile to resemble PRC2 occupancy in embryonic fibroblasts [133]. Most of the genes repressed by *HOTAIR*-induced PRC2 binding are involved in cell-cell signaling, metastasis and development [133]. Increased expression of *HOTAIR* is also observed in liver cancer tissues, and these patients exhibit increased risk of recurrence after hepatectomy and lymph node metastasis [134,135]. Other noncoding RNAs involved in liver cancer pathogenesis are described in a recent and comprehensive review [136]. Zhang et al. showed that PLK1-dependent ubiquitination of SUZ12 is enhanced by overexpression of *HOTAIR* [56]. *HOTAIR* acts as the RNA scaffold that forms the PRC2/DDX5 complex. *HOTAIR* also serves as a scaffold for ubiquitination, binding the E3 ubiquitin ligase Mex3B [137]. DDX5 displaces Mex3B from *HOTAIR* as a result of which SUZ12 is stabilized. Upon downregulation of DDX5, Mex3B binds *HOTAIR* and promotes SUZ12 ubiquitination [42,56]. Also, HCC patients with at least 2-fold increased expression of PLK1 and *HOTAIR*, show increased *EPCAM* expression, a PRC2 target gene [42,56].

## 10. Summary and Future Directions

**Figure 3:**
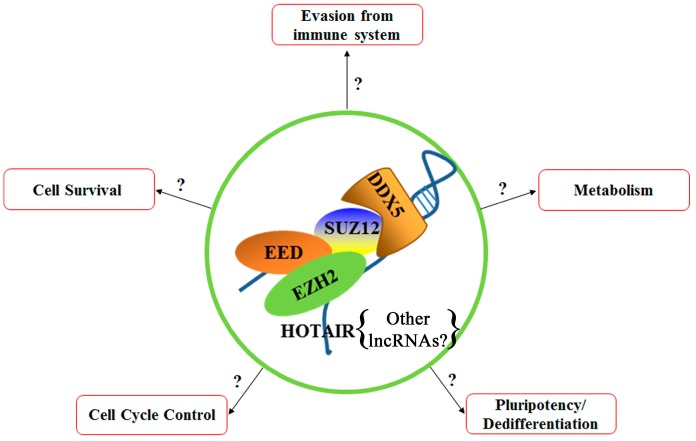
Proposed model: DDX5 in association with the PRC2 complex could coordinate via epigenetic mechanisms expression of different cascades of signaling pathways that ultimately could bring about the hCSC phenotype. The relevant pathways are shown.

Our laboratory has identified several important molecules involved in the development of poor prognosis HCC due to chronic HBV infection. These include: the RNA helicase DDX5 that stabilizes the PRC2 complex in association with the lncRNA *HOTAIR* to repress transcription of select cellular genes. Upon downregulation of DDX5 by HBV infection, as it has been observed in a subgroup of poor prognosis HBV-related HCC, the SUZ12 subunit of PRC2 undergoes proteasomal degradation. In turn, loss of PRC2 function enables re-expression of select PRC2 repressed genes, including *EPCAM* and pluripotency genes. Current efforts (Figure 3) are directed towards understanding whether DDX5 alone or in association with PRC2 orchestrates expression of pluripotency genes together with genes linked to cell survival, rescue from p53-independent regulated cell death, and metabolism-related genes to reprogram differentiated hepatocytes to a hCSC phenotype. Additional questions that need to be further investigated include the role of other non-coding RNAs in this epigenetic mechanism, as well as the role of this epigenetic mechanism in virus biosynthesis. 

## Figures and Tables

**Table 1:** Genes and Proteins encoded by Hepatitis B Virus (HBV).

Gene	Protein
P	Reverse transcriptase/DNA polymerase (Pol)
X	HBx protein
C	Capsid protein/Core antigen (HBcAg)
S	Surface/Envelope antigen (HBsAg)

**Table 2:** Epigenetic Modifications and Regulators in Hepatocellular Carcinoma (HCC).

Epigenetic Modification	Epigenetic Regulators
Global hypomethylation, promoter hypermethylation of tumor suppressor and anti-proliferative genes	DNA methyltransferases DNMT1, DNMT3A and DNMT3B over-expressed[48,49]
miRNA and Lnc RNA mis-expression	Downregulated: miR-26[50], miR-195, miR-199a, miR-200a, miR-125a, miR-122[51] Upregulated: miR-224, miR-106b~25, miR-17~92, miR-18 [52]
Histone modifications	Histone deacetylases: overexpression of HDAC1, HDAC2 and HDAC3 [53]. Overexpression of EZH2 [54] and proteasomal degradation of SUZ12 [55,56], both core subunits of PRC2.
Nucleosome re-structuring	Mutations in subunits of SWI/SNF (ARID1A, ARID1B) [57], poly-bromo and BRG1-associated (PBAF) remodeling complex (ARID2) [58]
